# Nasopharyngeal Staphylococcus aureus Isolation and Bacterial Culture Profiles in COVID-19 Patients: A Comparative Study Based on Lung Involvement

**DOI:** 10.7759/cureus.87149

**Published:** 2025-07-02

**Authors:** Burcu Vural Camalan, Naciye Badir, Sumeyra Doluoglu, Onural Ozturk

**Affiliations:** 1 Department of Otorhinolaryngology Head and Neck Surgery, University of Health Sciences Ankara Etlik City Hospital, Ankara, TUR; 2 Department of Medical Microbiology, Simav Doc. Dr. Ismail Karakuyu State Hospital, Kutahya, TUR; 3 Department of Radiology, Faculty of Medicine, Northwestern University Feinberg School of Medicine, Chicago, USA

**Keywords:** bacterial colonization, covid-19, lung involvement, nasopharyngeal culture, staphylococcus aureus

## Abstract

Purpose: This exploratory study aimed to determine the nasopharyngeal carriage rate of *Staphylococcus aureus *and to characterize the distribution of other culturable bacterial species in patients with COVID-19. Additionally, we assessed whether bacterial growth patterns differed between patients with and without lung involvement.

Methods: Nasopharyngeal samples were collected from COVID-19 patients with and without lung involvement, as assessed by thoracic CT scans. The study also included PCR-negative control patients. Sampling occurred between January and March 2021 at a state hospital outpatient clinic. A total of 65 participants were included in the study: 51 (78.5%) tested positive for COVID-19, of whom 25 (49.0%) had lung involvement and 26 (51.0%) did not. The remaining 14 (21.5%) served as PCR-negative controls.

Results: *S. aureus* was isolated in 11 (21.6%) of COVID-19-positive patients and 5 (35.7%) of controls. Among COVID-19 patients, the isolation rate was 6 (24.0%) in those with lung involvement and 5 (19.2%) in those without. No statistically significant differences were observed in *S. aureus* carriage or in the overall bacterial profiles across the groups.

Conclusion: Nasopharyngeal *S. aureus *colonization and bacterial distribution showed no significant association with COVID-19 status or lung involvement. Although colonization is common, its clinical relevance in early-stage COVID-19 remains unclear and warrants further study.

## Introduction

The nasopharynx plays a pivotal role in the progression of respiratory tract infections, with its microbial balance composition serving as a key component of host defense [1]. Emerging evidence suggests that respiratory viruses can disrupt both upper and lower respiratory tract microbiota, promoting the proliferation of opportunistic pathogens and potentially influencing disease trajectory [2,3]. During the COVID-19 pandemic, numerous studies have explored how alterations in the nasopharyngeal microbiota may affect disease severity and clinical outcomes in infected individuals [4,5].

With recent developments in DNA sequencing technologies, the nasopharyngeal microbiota has been characterized in detail [6]. Nevertheless, traditional culture methods remain the gold standard for detecting culturable pathogenic bacteria, especially in diagnostic and treatment processes [7]. *Staphylococcus aureus*, which can be readily identified through nasopharyngeal culture, may exist as a commensal organism in healthy individuals or cause secondary bacterial infections during viral illnesses [8]. Recent insights into the biology of *S. aureus* have highlighted the critical role of metal ion acquisition systems, particularly under infection-associated stress. This pathogen employs highly coordinated molecular mechanisms, including the production of low-molecular-weight metal chelators known as metallophores, to overcome host-imposed nutritional immunity. These adaptive strategies support bacterial persistence, colonization, and evasion of immune responses in nutrient-limited environments such as the nasopharynx [9]. Some studies have reported that co-infection with *S. aureus* in patients with SARS-CoV-2 is associated with a higher mortality rate (61.7%) compared to SARS-CoV-2 infection alone [10]. However, the data on nasopharyngeal *S. aureus* isolation in COVID-19 patients remain limited in the literature.

This exploratory study aimed to determine the nasopharyngeal carriage rate of *S. aureus* and to characterize the distribution of other culturable bacterial species in patients with COVID-19. Additionally, we assessed whether bacterial growth patterns differed between patients with and without lung involvement.

## Materials and methods

This study was designed as a prospective study. Patients who presented to the pandemic outpatient clinic of Simav Doc. Dr. Ismail Karakuyu State Hospital, Kutahya, Turkey, between January and March 2021, were included. Ethical approval (No: 2021-01/05) was obtained from the Ethics Committee of Kutahya Health Sciences University, a tertiary care institution. Informed consent was obtained from all patients and volunteers. Nasopharyngeal swab samples were collected from COVID-19-positive patients and healthy controls. Individuals were excluded if they were under 18 years of age, had previously received the pneumococcal vaccine, had used antibiotics or been hospitalized within the past 30 days, or did not provide informed consent.

The study included 26 COVID-19-positive patients without radiographic evidence of pneumonia, 25 COVID-19-positive patients with CT findings consistent with COVID-19 pneumonia who were hospitalized, and 14 asymptomatic healthy volunteers with negative PCR results. Patients with suspected COVID-19 infection but no radiographic evidence of pneumonia were monitored at home following the standard treatment protocol. Nasopharyngeal swab samples were collected from these patients at home after confirmation of a positive PCR result during the monitoring period. The swab collection process did not interfere with the patients' routine follow-up or treatment. Nasopharyngeal swab samples were also obtained from asymptomatic healthy volunteers using sterile Stuart transport swabs. All samples were transported in Stuart transport medium (Becton Dickinson, Franklin Lakes, NJ, US).

Nasopharyngeal swab samples underwent direct Gram staining and traditional qualitative microbiological culture. Samples were inoculated onto 5% sheep blood agar, chocolate agar, and eosin methylene blue agar (Becton Dickinson, Franklin Lakes, NJ, US). The culture plates were then incubated aerobically at 37°C (eosin methylene blue agar) and 5% carbon (5% sheep blood and chocolate agar). Cultures were examined at 24 and 48 hours, and all distinct colony types were isolated. Both pathogenic and non-pathogenic Gram-negative and Gram-positive bacteria were analyzed. Bacterial identification was performed using the BD BBL™ Crystal™ Identification System (Becton Dickinson, Franklin Lakes, NJ, US), employing the Gram-Positive ID Kit for Gram-positive organisms and the Enteric/Nonfermenter ID Kit for Gram-negative organisms. Identification results were recorded and evaluated on a per-patient basis.

The presence of both normal nasopharyngeal flora and pathogenic bacteria was compared across study groups. The distribution of culturable bacteria was analyzed among COVID-19-positive patients with and without pneumonia and compared with that of COVID-19-negative individuals.

Categorical data were expressed as numbers (n) and percentages (%), while quantitative data were reported as mean ± SD or median (25th-75th percentiles), where applicable. The Shapiro-Wilk test was used to assess normality, and Levene’s test was applied to evaluate the homogeneity of variances. Group comparisons for normally distributed variables were performed using the Student’s t-test, while the Mann-Whitney U test was used for non-normally distributed data. Qualitative variables were analyzed using the continuity-corrected χ² test or Fisher’s exact test, as appropriate. All statistical analyses were conducted using IBM SPSS Statistics for Windows, Version 25.0 (Released 2017; IBM Corp., Armonk, NY, USA). A p-value < 0.05 was considered statistically significant.

## Results

The study included 65 participants with a mean age of 51.3 ± 18.5 years. Among them, 29 (44.6%) were male and 36 (55.4%) were female. Hypertension and diabetes mellitus were the most common comorbidities. Non-pathogenic bacterial growth was detected in 55 (84.6%) participants, while pathogenic bacterial growth was observed in 36 (55.4%). The demographic and clinical characteristics of all participants are presented in Table 1.

**Table 1:** Demographic and clinical characteristics of all patients included in the study Descriptive statistics were expressed as mean ± standard deviation (*) or median (**; 25th-75th percentile).

Variables	Value
Age (years)*	51.3±18.5
Age range (years)	19-87
Sex	
Male	29 (44.6%)
Female	36 (55.4%)
Comorbidities	
Hypertension	19 (29.2%)
Diabetes mellitus	8 (12.3%)
Asthma	3 (4.6%)
Heart failure	2 (3.1%)
Chronic renal failure	2 (3.1%)
Time to nasopharyngeal culture (days)**	5.0 (3.0-8.0)
Non-pathogenic growth	55 (84.6%)
Pathogenic growth	36 (55.4%)
Mortality	5 (7.7%)

When comparing COVID-19-positive patients (n = 51) with COVID-19-negative controls (n = 14), no significant differences were observed in terms of age, sex, or the rates of pathogenic and non-pathogenic bacterial growth (p > 0.05 for all comparisons). A detailed comparison between groups is presented in Table 2.

**Table 2:** Comparison of demographic and clinical characteristics of patients based on COVID-19 status ^†^Student's t-test. ^‡^χ^2^ test with continuity correction. ^¶^Fisher's exact probability test.

Variables	COVID-19-negative (n = 14)	COVID-19-positive (n = 51)	p-value
Age (years)	46.6 ± 17.5	52.7 ± 18.7	0.291^†^
Sex			0.447^‡^
Male	8 (57.1%)	21 (41.2%)	
Female	6 (42.9%)	30 (58.8%)	
Non-pathogenic growth	13 (92.9%)	42 (82.4%)	0.676^¶^
Pathogenic growth	9 (64.3%)	27 (52.9%)	0.651^‡^
Mortality	0 (0.0%)	5 (9.8%)	0.576^¶^

Regarding the distribution of isolated microorganisms, *coagulase-negative staphylococci* were the most commonly detected non-pathogenic organisms in both groups. *Corynebacterium *spp. were identified in 12 patients (23.5%) in the COVID-19-positive group but were not identified in any COVID-19-negative participants. Among the pathogenic bacteria, *S. aureus* was the most frequently isolated species, followed by *Enterococcus *spp. and *Klebsiella pneumoniae*. A detailed breakdown of bacterial species identified in the COVID-19-positive and COVID-19-negative groups is presented in Table 3.

**Table 3:** Distribution of bacterial species isolated from COVID-19-positive and COVID-19-negative subjects

Bacterial species	COVID-19-negative (n = 14)	COVID-19-positive (n = 51)	Total (n = 65)
Non-pathogenic			
Coagulase-negative staphylococci	11 (78.6%)	40 (78.4%)	51 (78.5%)
*Corynebacterium* spp.	0 (0.0%)	12 (23.5%)	12 (18.5%)
Streptococcus viridans	5 (35.7%)	16 (31.4%)	21 (32.3%)
*Micrococcus *spp.	1 (7.1%)	3 (5.9%)	4 (6.2%)
Pathogenic			
Staphylococcus aureus	5 (35.7%)	11 (21.6%)	16 (24.6%)
*Enterococcus* spp.	6 (42.9%)	7 (13.7%)	13 (20.0%)
*Bacillus *spp.	0 (0.0%)	1 (2.0%)	1 (1.5%)
Escherichia coli	0 (0.0%)	3 (5.9%)	3 (4.6%)
Klebsiella oxytoca	1 (7.1%)	2 (3.9%)	3 (4.6%)
*Morganella *spp.	0 (0.0%)	2 (3.9%)	2 (3.1%)
Klebsiella pneumoniae	0 (0.0%)	6 (11.8%)	6 (9.2%)
Haemophilus influenzae	0 (0.0%)	1 (2.0%)	1 (1.5%)
Streptococcus pneumoniae	2 (14.3%)	3 (5.9%)	5 (7.7%)

Within the COVID-19-positive group, patients were further stratified based on lung involvement as confirmed by chest CT imaging. As shown in Table 4, patients with lung involvement were significantly older than those without (65.2 ± 15.7 years vs. 40.4 ± 12.1 years; p < 0.001). The mortality rate was also significantly higher in patients with lung involvement (5, 20.0%) compared to those without (0, 0.0%) (p = 0.023). However, there were no significant differences between the subgroups in terms of pathogenic or non-pathogenic bacterial growth (p > 0.05 for both). Table 5 presents the detailed distribution of cultured bacteria based on lung involvement status. 

**Table 4:** Comparison of demographic and clinical characteristics of patients according to lung involvement ^†^Student's t-test. ^‡^χ^2^ test with continuity correction. ^¶^Fisher's exact probability test.

Variables	No CT involvement (n = 26)	CT involvement (n = 25)	p-value
Age (years)	40.4 ± 12.1	65.2 ± 15.7	<0.001^†^
Sex			0.907^‡^
Male	10 (38.5%)	11 (44.0%)	
Female	16 (61.5%)	14 (56.0%)	
Non-pathogenic growth	21 (80.8%)	21 (84.0%)	>0.999^¶^
Pathogenic growth	13 (50.0%)	14 (56.0%)	0.882^‡^
Mortality	0 (0.0%)	5 (20.0%)	0.023^¶^

**Table 5:** Nasopharyngeal culture results according to lung involvement in COVID-19-positive patients

Bacterial species	No CT involvement (n = 26)	CT involvement (n = 25)
Non-pathogenic		
Coagulase-negative staphylococci	20 (76.9%)	20 (80.0%)
*Corynebacterium* spp.	4 (15.4%)	8 (32.0%)
Streptococcus viridans	7 (26.9%)	9 (36.0%)
*Micrococcus* spp.	0 (0.0%)	3 (12.0%)
Pathogenic		
Staphylococcus aureus	5 (19.2%)	6 (24.0%)
*Enterococcus *spp.	1 (3.8%)	6 (24.0%)
*Bacillus* spp.	1 (3.8%)	0 (0.0%)
Escherichia coli	0 (0.0%)	3 (12.0%)
Klebsiella oxytoca	1 (3.8%)	1 (4.0%)
*Morganella* spp.	1 (3.8%)	1 (4.0%)
Klebsiella pneumoniae	3 (11.5%)	3 (12.0%)
Haemophilus influenzae	1 (3.8%)	0 (0.0%)
Streptococcus pneumoniae	3 (11.5%)	0 (0.0%)

*S. aureus* was identified in 11 (21.6%) of the COVID-19-positive patients. When stratified by lung involvement, it was isolated in six patients (24.0%) with lung involvement and five (19.2%) without. Among the COVID-19-negative individuals, *S. aureus* was isolated in 5 (35.7%) of 14 patients (Table 6).

**Table 6:** Comparison of Staphylococcus aureus isolation ^¶^Fisher's exact probability test.

Subjects	*Staphylococcus aureus-*positive	*Staphylococcus aureus-*negative	p-value
COVID-19-negative (n = 14)	5 (35.7%)	9 (64.3%)	0.471^¶^
COVID-19-positive (n = 51)	11 (21.6%)	40 (78.4%)	
No CT involvement (n = 26)	5 (19.2%)	21 (80.8%)	0.730^¶^
CT involvement (n = 25)	6 (24.0%)	19 (76.0%)	

## Discussion

The nasopharynx serves as both an entry point for respiratory tract infections and a critical site for initiating immune defense. The nasopharyngeal microbiota comprises a community of commensal microorganisms that enhance host immunity, support the epithelial barrier, and regulate mucosal immune responses [1]. Maintaining a balanced microbiota helps prevent pathogen colonization and supports overall respiratory health. This microbial community also interacts with nasopharyngeal lymphoid tissue, contributing to the development of both innate and adaptive immune responses [2,11,12]. Previous studies have linked alterations in the composition of the nasopharyngeal microbiota to increased susceptibility and severity of viral infections, including influenza A and B, rhinoviruses, and respiratory syncytial virus [13]. Since the emergence of the COVID-19 pandemic, the potential impact of SARS-CoV-2 on the nasopharyngeal microbiota, and whether such changes influence disease prognosis, has become a subject of growing interest [14,15]. In this study, we aimed to characterize the culturable bacterial component of the nasopharyngeal microbiota in COVID-19 patients, with a particular focus on the isolation of *S. aureus* in nasopharyngeal cultures, and to investigate whether bacterial isolation differed between patients with and without lung involvement.

There is marked inconsistency in the literature regarding the relationship between nasopharyngeal microbiome diversity and COVID-19 severity. Some studies have reported an increased abundance of pathogenic bacteria in the nasopharynx of patients with more severe COVID-19. For example, Llorens-Rico et al. observed a dominance of *Staphylococcus* and *Corynebacterium* in ICU-admitted patients, although no control group was included [16]. Similarly, Bai et al. found a higher prevalence of pathogenic bacteria and a reduction in commensals in critically ill patients compared to uninfected controls [17]. Qin et al. reported that *Staphylococcus*, *Corynebacterium*, and *Acinetobacter* were isolated in 40.7% of severe cases versus 10.8% of mild ones [18]. Giugliano et al. also identified a correlation between high viral burden and the presence of *S. aureus, K. pneumoniae, S. pneumoniae*, and other super-pathogens [19]. Conversely, other studies have observed a reduction in pathogenic bacteria in more severe cases. Chen et al. demonstrated a lower abundance of opportunistic pathogens such as *Actinomyces*, *Prevotella*, and *Veillonella *in patients with severe COVID-19 compared to those with milder forms [20]. Additionally, studies by Shilts et al. and la fortune Tchoupou Saha et al. found that *Corynebacterium* abundance decreased as disease severity increased [4,5]. Meanwhile, a third group of studies reported no significant differences in the nasopharyngeal microbiota composition between asymptomatic SARS-CoV-2-positive individuals and healthy controls [4,21,22]. Similarly, in our study, although various culturable bacteria were isolated from nasopharyngeal samples, including both commensals and potential pathogens, no statistically significant differences in bacterial isolation rates were found between COVID-19-positive and COVID-19-negative groups, nor between patients with and without lung involvement. This variability across studies underscores the absence of a clear consensus and highlights the complex interplay between host immunity, microbial communities, and viral infections.

In our study, *S. aureus* was isolated from the nasopharyngeal swabs of 11 (21.6%) COVID-19-positive patients and 5 (35.7%) COVID-19-negative individuals. Among the COVID-19-positive patients, *S. aureus* was detected in 6 (24.0%) patients with lung involvement and 5 (19.2%) without. However, none of these differences were statistically significant (p = 0.471 and p = 0.730, respectively). These findings suggest that *S. aureus* colonization is common and may not be directly associated with COVID-19 severity. Previous studies have linked *S. aureus* co-infection to worse clinical outcomes, especially in hospitalized patients [10]. Piechowicz et al. further reported that *S. aureus* strains isolated from COVID-19 patients may exhibit increased virulence [23]. However, since cultures in our study were collected prior to hospitalization, they reflect community-based colonization rather than hospital-acquired infection. While this limits direct comparison with inpatient settings, early detection of *S. aureus *colonization through nasopharyngeal culture may still hold prognostic value. Controlled studies are needed to further clarify this relationship.

Additionally, our study found that the average age of patients with lung involvement was significantly higher than those without. This aligns with the well-established evidence that age is a major risk factor for severe COVID-19 [24]. The age-related decline in immune function and increased susceptibility to respiratory complications likely contribute to this observation.

This study presents nasopharyngeal S. aureus isolation rates in patients with COVID-19 and offers a descriptive overview of the culturable bacterial components of the nasopharyngeal microbiota. The comparison of nasopharyngeal culture results among COVID-19-positive patients with and without lung involvement and healthy controls revealed no statistically significant differences between the groups.

Nevertheless, several limitations should be acknowledged. The sample size was relatively small, which may have limited the statistical power of our findings. Additionally, the use of conventional culture methods restricted the analysis to organisms that grow under standard laboratory conditions, thereby excluding many anaerobic and fastidious bacteria that are better characterized using sequencing-based approaches. As a promising alternative, culturomics has emerged as a high-throughput, culture-based methodology that employs a broad range of growth conditions to enhance microbial recovery. This technique enables the detection of rare, slow-growing, or previously uncultured microorganisms, substantially expanding the known microbial repertoire beyond the capabilities of conventional culture or sequencing alone [25]. Furthermore, due to the cross-sectional nature of this study, dynamic changes in nasopharyngeal bacterial composition over time could not be assessed.

## Conclusions

This study investigated the nasopharyngeal colonization patterns of culturable bacteria in both COVID-19-positive patients and COVID-19-negative controls and evaluated their potential association with lung involvement among COVID-19 cases. *S. aureus *was the most frequently identified pathogen, followed by *Enterococcus* spp. and* K. pneumoniae*. Although no statistically significant differences in bacterial growth were observed between COVID-19-positive and COVID-19-negative groups, or between patients with and without lung involvement, several clinically relevant observations were noted. Patients with lung involvement were significantly older and had higher mortality rates. Among COVID-19-positive individuals, the overall *S. aureus* isolation rate was 21.6%, with a slightly higher prevalence in those with lung involvement (24.0%) compared to those without (19.2%). In COVID-19-negative individuals, *S. aureus* was isolated in 35.7% of cases. These findings provide insight into colonization patterns during the early phase of infection. While colonization appears common, its clinical significance in early-stage COVID-19 warrants further investigation.
